# Observation of Chronic Graft-Versus-Host Disease Mouse Model Cornea with In Vivo Confocal Microscopy

**DOI:** 10.3390/diagnostics11081515

**Published:** 2021-08-23

**Authors:** Shota Shimizu, Shinri Sato, Hiroko Taniguchi, Eisuke Shimizu, Jingliang He, Shunsuke Hayashi, Kazuno Negishi, Yoko Ogawa, Shigeto Shimmura

**Affiliations:** 1 Department of Ophthalmology, Keio University School of Medicine, Tokyo 160-8582, Japan; shimisho@keio.jp (S.S.); tani@keio.jp (H.T.); ophthalmolog1st.acek39@keio.jp (E.S.); hejingliangai@126.com (J.H.); shun.hayashi8840@keio.jp (S.H.); kazunonegishi@keio.jp (K.N.); shige.shimmura@keio.jp (S.S.); 2 Eye Center, The Second Affiliated Hospital of Zhejiang University School of Medicine, Hangzhou 310003, China

**Keywords:** hematopoietic stem cell transplantation, bone marrow transplantation, graft-versus-host disease, dry eye disease, in vivo confocal microscopy

## Abstract

Graft-versus-host disease (GVHD) is a major complication after hematopoietic stem cell transplantation (HSCT), and ocular GVHD can cause severe dry eye disease that can lead to visual impairment. Epithelial damage, vascular invasion, corneal fibrosis, and corneal perforation may occur in severe cases. It is generally accepted that inflammatory cells such as dendritic cells and T cells contribute to this pathological condition. However, it is still unknown what pathological condition occurs on the ocular surface after HSCT, and when. We therefore observed the dynamics of inflammatory cells in the cornea of chronic GVHD (cGVHD) model mice from 1 to 4 weeks after bone marrow transplantation (BMT) by in vivo confocal microscopy (IVCM) and considered the relationship with the pathophysiology of ocular GVHD (tear volume, corneal epithelial damage). In the allogeneic group, neovascularization occurred in all eyes at 1 week after BMT, although almost all vessels disappeared at 2 weeks after BMT. In addition, we revealed that infiltration of globular cells, and tortuosity and branching of nerves in the cornea occurred in both cGVHD mice and human cGVHD patients. Thus, we consider that cGVHD mouse model study by IVCM reproduces the state of ocular GVHD and may contribute to elucidating the pathological mechanism for ocular GVHD.

## 1. Introduction

Hematopoietic stem cell transplantation (HSCT) has been established as an effective treatment for hematological malignancies and the number of HSCTs is increasing year by year [1]. However, graft-versus-host disease (GVHD), an immune response that occurs between the donor graft and the recipient’s cells or tissues, may occur after HSCT. Traditionally, GVHD is divided into acute GVHD (aGVHD) and chronic GVHD (cGVHD) by the onset, aGVHD occurring less than 100 days and cGVHD occurring more than 100 days after bone marrow transplantation (BMT) [2,3]. However, recently, early cGVHD and late aGVHD appeared along with diversification of HSCT techniques, and the classification of GVHD was changed, with more focus on clinical symptoms. The National Institute of Health (NIH) Consensus Conference newly defined two main categories of GVHD, each with two subcategories [1,4]. In this definition, aGVHD includes classic aGVHD and persistent, recurrent, or late-onset aGVHD occurring more than 100 days after HSCT. On the other hand, cGVHD includes classic cGVHD and overlap syndrome which is defined as a combination of classic cGVHD and one or more aGVHD symptoms [5].

The pathophysiology of aGVHD is as follows. Irradiation and anti-cancer drugs prior to HSCT damage the tissues of the skin, liver, and gastrointestinal mucosa and cause inflammation in HSCT recipients. Then, the donor-derived T cells activate and further damaged these target tissues. As a result, rash, jaundice, and diarrhea occur. On the other hand, the pathophysiology of cGVHD is complex because it involves not only T cell-derived cell-mediated immunity but also B-cell-derived humoral immunity, in addition to immune-mediated fibrosis [1,4]. Thus, the symptoms are more diverse than those of acute GVHD. The target organs are the eyes, oral cavity, lungs, skin, intestine, liver and muscular/skeletal system. cGVHD occurs in 40–60% of patients undergoing HSCT [6,7]. Ocular signs include dry eye disease (DED), conjunctivitis, corneal epithelial damage, vascular invasion and fibrosis in the ocular surface and lacrimal glands, meibomian gland dysfunction, and in severe cases, corneal perforation [8,9]. DED is the most common post-transplant ophthalmic complication and is estimated to occur in about 50–60% of HSCT cases [10]. cGVHD-related DED often progresses to intractable condition [11]. There have been many attempts to elucidate the processes leading to the onset and progression of the pathological condition and to establish better treatment methods.

In our study, we mainly used in vivo confocal microscopy (IVCM) to investigate cGVHD mouse cornea. IVCM emits a 670 nm wavelength diode laser as a light source to focus on a specific surface in a thick sample while simultaneously excluding extra reflected light from the unfocused top and bottom surfaces. Thus, a clear image with a high contrast ratio can be obtained only on a certain focal plane [12]. A clearer and higher resolution cross-sectional image can be obtained at the cellular level. As a result, it is suitable to localize various cells and for grasping their shape. In addition, it is possible to comprehend the progress of the pathological condition and any therapeutic effects because IVCM can repeatedly observe the same point in the cornea without invasion [13]. Furthermore, recent studies have investigated the utility of an artificial intelligence-based deep learning algorithm with IVCM. It is expected that the analysis of the IVCM image will become faster and more accurate for clinical screening using artificial intelligence [14].

Infiltration of small round lymphocytes in the cornea of ocular GVHD patients has been reported and it was shown that these inflammatory cells contribute to the pathology of cGVHD [15]. However, it is still unclear when and what immune cells infiltrate after BMT. We therefore observed the cornea of a cGVHD mouse model every week after BMT by IVCM. Simultaneously, we evaluated changes in tear volume, corneal epithelial damage by fluorescein staining, and pathological changes in the lacrimal gland. Then, we considered correlations of the results and the condition of GVHD-related DED.

In this study, we used IVCM to observe the time-course changes of the cornea in the cGVHD mouse model after BMT. Infiltration of small round cells and dendritic cells and tortuosity and branching of nerves in the cornea of GVHD mice were observed in the allogeneic group and similar findings could be seen in the cornea of human cGVHD observed by confocal microscopy in a clinical setting. Consequently, we found that the analyses for cGVHD mouse model by IVCM were effective as a tool for elucidating the pathophysiology of ocular GVHD.

## 2. Materials and Methods

### 2.1. Mice

BALB/c mice (8 week-old) and B10.D2 (8 week-old) were obtained from Sankyo Laboratory, Inc. (Tokyo, Japan). Our protocols for animal experiments were approved by the Keio University Institutional Animal Care and Use Committee (# 09152) and we followed the ARVO Statement for the Use of Animals in Ophthalmic and Vision Research. All experimental procedures were in accordance with the Institutional Guidelines on Animal Experimentation at Keio University.

### 2.2. Bone Marrow Transplantation

To create the cGVHD mouse model, 8 week-old male B10.D2 and female BALB/c mice were each used as donors and recipients for allogeneic bone marrow transplantation (BMT). As a non cGVHD control, syngeneic BMT was conducted by transplanting donor cells from male BALB/c mice to female BALB/c mice. The recipient mice were irradiated with X-ray (7Gy) using a Gammacell 137Cs source (Hitachi Medico, Ltd., Tokyo, Japan) prior to BMT. The femurs, tibias and spleens from donor mice were harvested. The bones were gently crushed manually using a sterile stick, and bone marrow (BM) cells were released by mixing the fragments of bones with Roswell Park Memorial Institute (RPMI) medium. The suspension of BM cells was filtered to remove the impurities. The spleens were also gently crushed into a cell suspension and filtered. Ammonium-Chloride-Potassium (ACK) lysing buffer (Quality Biological, Inc., Gaithersburg, MD, USA) was used to lyse the red blood cells in splenic cells. The BM cells and splenic cells with RPMI medium were suspended and adjusted the density of cells to 1 × 10^6^/100 μL and 2 × 10^6^/100 μL, respectively. A mixture consisting of 100 μL of BM cell suspension and 100 μL of splenic cell suspension was prepared and injected into each recipient mouse per 200 µL through the tail vein. We added splenic cells for the enrichment of mature T cells according to the methods of Zhang et al. [16].

### 2.3. In Vivo Confocal Microscopy

**Figure 1:**
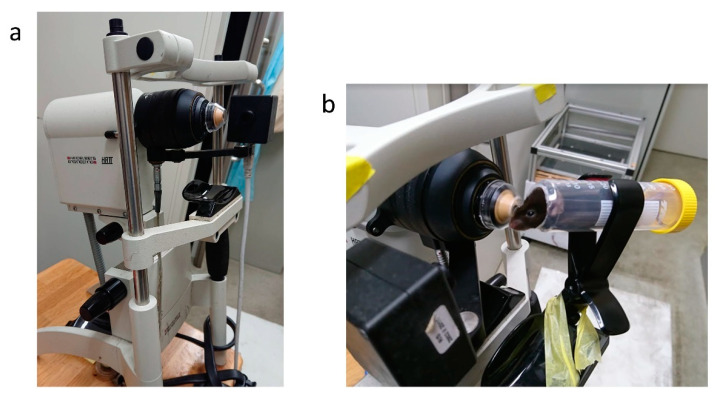
Cornea-specific in vivo laser confocal microscopy for mouse: (**a**) Heidelberg Retina Tomograph 2 Rostock Cornea Module (HRT2-RCM); (**b**) Mouse fixing device.

The cornea of all mice and patients was examined by in vivo confocal laser scanning microscopy (IVCM) (the Rostock Corneal Software, ver. 1.2, Heidelberg Retina Tomograph II [RCM/HRT II]; Heidelberg Engineering GmbH) (Figure 1a). A drop of gel (Bausch & Lomb, GmbH, Berlin, Germany) was placed on the top of the object lens and the anterior surface of a poly-methyl-methacrylate (PMMA) cap (Tomo-cap; Heidelberg Engineering GmbH) as a coupling medium. The examiner placed the PMMA cap onto the area of interest, and was examined through real-time video images displayed on a computer screen. The target area and depth were adjusted manually and images (400 × 400 μm in size) were captured. Images of neovascularization and nerve are similar on IVCM still images; however, vessels were identified by the movement of blood cells in the vascular cavity on live feeds (Supplementary Video S1).

At the beginning of the examination, each animal was anesthetized by intramuscular injection of a mixture of 4 mg/kg midazolam, 0.75 mg/kg medetomidine, and 5 mg/kg butorphanol. We made a device for fixing the mouse and efficiently imaging the cornea (Figure 1b). The 2 mL scale portion of the 50 mL syringe was cut horizontally. Then, we put the anesthetized mouse in the syringe and fixed it in the clip for fixing a smartphone. The head of the mouse could be fixed by the opened hole at the top of the syringe. While operating this machine, the mouse adjusted the focal plane by this device to ensure that all layers of each target area were clearly recorded. Lastly, each mouse was anti-anesthetized by intramuscular injection of 0.75 mg/kg atipamezole.

Meanwhile, the topical anesthetic 0.4% oxy-buprocaine was administered for cGVHD patients to their conjunctival sac at the beginning of examination. The patients were instructed to place their heads and chins in holders and continue looking forward (during examination of the central cornea) or looking up (during examination of the inferior cornea).

### 2.4. Fluorescein Staining on the Ocular Surface

Corneal fluorescein staining (CFS) was performed in order to assess the degree of corneal epithelial damage every week until 4 weeks after BMT. One μL of 0.5% fluorescein sodium solution (Fluorescite, 877290, Novartis Pharma, Tokyo, Japan) was dropped on the center of cornea. The ocular surface was observed by a Smart Eye Camera (13B2X10198030101, OUI Inc., Tokyo, Japan) under cobalt blue light [17]. The CFS value was calculated 90 s after fluorescein instillation. Each cornea was divided into three areas and each area was scored individually. The CFS value was scored based on a 0 to 4 grading scale: 0, absent; 1, slightly punctate staining with <30 spots; 2, punctate staining with >30 spots but not diffuse; 3, severe diffuse staining but no positive plaques; and 4, positive fluorescein plaques. The scores obtained from the three areas were summed to obtain a final grade (0–12 points) [18,19].

### 2.5. Cotton Thread Test

Tear volume was measured by inserting a phenol red thread (Zone-Quick; 2564187, Showa Yakuhin Kako Co., Ltd., Tokyo, Japan) into the lateral canthus for 15 s. The length of the thread turned red was measured for both eyes, and the average value was used to evaluate the tear volume [20].

### 2.6. Cell Count in the Cornea

The number of dendritic cells and globular cells were counted in a circle with a diameter of 1000 µm from the center of the cornea in the syngeneic group and allogeneic group. A large area was analyzed since the variation of cell distribution in the peripheral cornea was larger than the central area.

### 2.7. Histological Analysis

The syngeneic and allogeneic-BMT recipient mice were sacrificed 3 and 4 weeks after the completion of BMT. The cornea and lacrimal glands of each mouse were harvested and fixed in 10% neutral buffered formalin for 2 h at room temperature, embedded in paraffin wax, and processed for Hematoxylin & Eosin (HE) and Mallory staining for cornea and lacrimal glands [21,22].

### 2.8. cGVHD Patients

This clinical study was approved by the Institutional Review Board and Ethics Committee of Keio Hospital (IRB No.: 20130013 and 20170350) and complied with the tenets of the Declaration of Helsinki. The individual participants were notified of all possible consequences of this study and gave written informed consent (No.: 20130013).

The GVHD group had 12 HSCT recipients with GVHD-related DED who survived for more than 100 days after BMT and the non-GVHD group had 10 recipients without GVHD-related DED. All patients were 20 years or older when they were recruited. The subjects in the GVHD group adapted the Japanese diagnostic criteria for DED. The details of this diagnostic criteria have been reported previously [13].

### 2.9. Statistical Analysis

Tailed *p*-values < 0.05. Statistical analysis was performed using GraphPad Prism 7.0.

## 3. Results

### 3.1. The Observation of Mouse Cornea by IVCM

#### 3.1.1. Serial Changes of Corneal Findings Observed by In Vivo Confocal Microscopy

**Figure 2:**
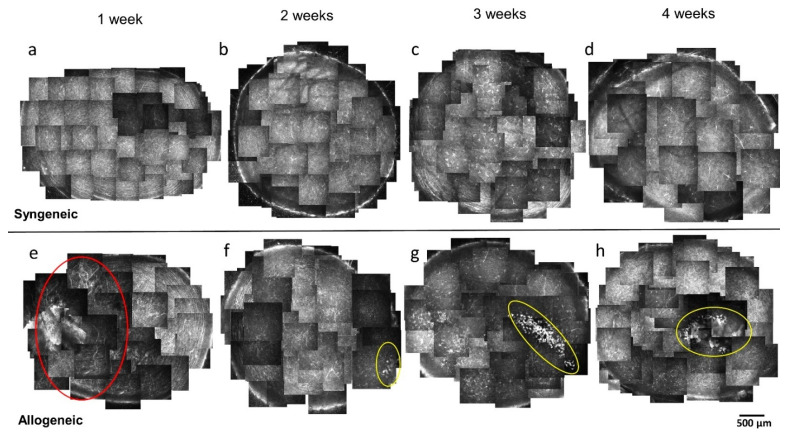
Time course in in vivo confocal microscopy (IVCM) images of cornea of the syngeneic group and allogeneic group: Upper side: (**a**–**d**) Representative IVCM images of the syngeneic group every week from 1 to 4 weeks; Lower side: (**e**–**h**) Representative IVCM images of the allogeneic group from 1 to 4 weeks, (**e**) Neovascularization (red circle area), (**f**–**h**) Activation of keratocytes (yellow circle area). Scale bar = 500 µm.

**Figure 3:**
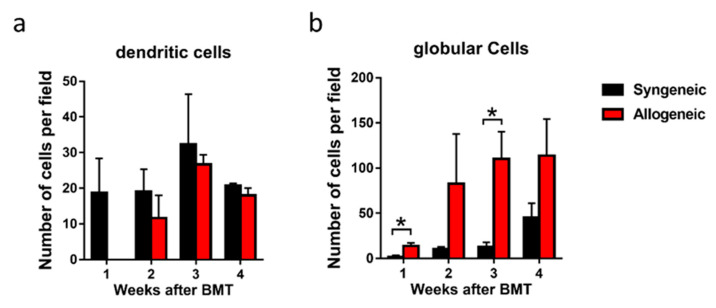
The number of cells in a circle with a diameter of 1000 µm from the center of the cornea in syngeneic group and allogeneic group: (**a**) The number of dendritic cells of syngeneic group and allogeneic group from 1 to 4 weeks after bone marrow transplantation (BMT); (**b**) The number of globular cells of syngeneic group and allogeneic group from 1 to 4 weeks after bone marrow transplantation. Data are presented as mean ± SEM. * *p* < 0.05, unpaired Student’s t-test. (*n* = 3 per group).

Serial changes of syngeneic group 1, 2, 3 and 4 weeks after BMT (Figure 2a–d), and allogeneic group 1, 2, 3 and 4 weeks after BMT (Figure 2e–h) are shown in Figure 2. In the syngeneic group, a significant difference was not observed during 1–4 weeks after BMT (Figure 2a–d). The presence of dendritic cells that were originally resident in the cornea became more prominent and enlarged 3 and 4 weeks after syngeneic BMT (Figure 2c,d). However, the number of dendritic cells did not change significantly between 2 to 4 weeks, though no dendritic cells were observed 1 week after allogeneic BMT (Figure 3a). The number of dendritic cells in the cornea was not significantly different in each week after both syngeneic and allogeneic BMT (Figure 3a). While the number of globular cells did not change significantly between 1 to 4 weeks after syngeneic BMT (Figure 3b), the number of globular cells in the cornea is higher in the allogeneic group than that of the syngeneic group through 1 to 4 weeks after BMT (Figure 3b). In addition, we detected bright areas which indicated activation of keratocyte 2 weeks after allogeneic BMT (Figure 2f–h), but not syngeneic BMT (Figure 2a–d).

#### 3.1.2. Neovascularization

**Figure 4:**
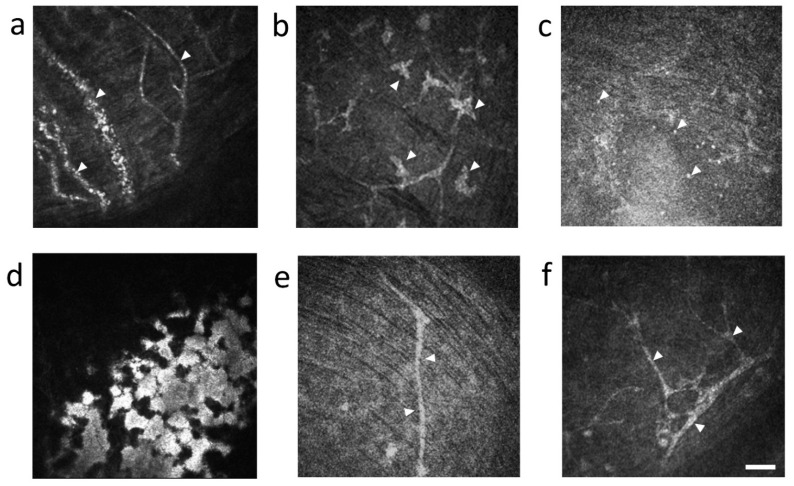
Representative in vivo confocal microscopic corneal images in mice: (**a**) neovascularization with moving blood cells in the allogeneic group 1 week after bone marrow transplantation (BMT) (arrowhead) and Supplementary Video S1; (**b**) dendritic cells with typical branching dendrites in the syngeneic group 3 weeks after bone marrow transplantation (BMT) (arrowhead); (**c**) small and globular cells in the allogeneic group 4 weeks after BMT (arrowhead); (**d**) activation of keratocyte in the allogeneic group 4 weeks after BMT; (**e**) nerve fibers with no abnormality in syngeneic group 4 weeks after BMT (arrowhead); (**f**) nerve fibers with tortuosity and branching in allogeneic group 4 weeks after BMT (arrowhead). Scale bar = 50 µm.

Neovascularization was not observed in the syngeneic group (Figure 2a–d), but was observed 1 week after BMT in the allogeneic group (Figure 2e and Figure 4a, Supplementary Figure S1). We could see blood cell movement in vessels (Supplementary Video S1). One week after BMT, neovascularization from the limbus to the center of the corneas were observed in all six corneas from three mice (Figure 2e) but regressed 2 weeks after allogeneic BMT (Figure 2f–h).

#### 3.1.3. Characteristic Cell Types in the Cornea

We observed dendritic cells with typical branching dendrites in the syngeneic and the allogeneic group (Figure 4b). On the other hand, in the allogeneic group, we observed small and globular cells (Figure 4c) and activation of keratocytes in subepithelial corneal layer, approximately 30 µm from the corneal surface (Figure 4d).

#### 3.1.4. The Change of Nerve Fibers

Nerve fibers did not change significantly in the syngeneic group (Figure 2a–d and Figure 4e). However, the tortuosity and branching of corneal nerves were observed 4 weeks after BMT in the allogeneic group (Figure 2e,f and Figure 4f).

### 3.2. Serial Changes of Corneal Fluorescein Staining and Tear Fluid Volume

**Figure 5:**
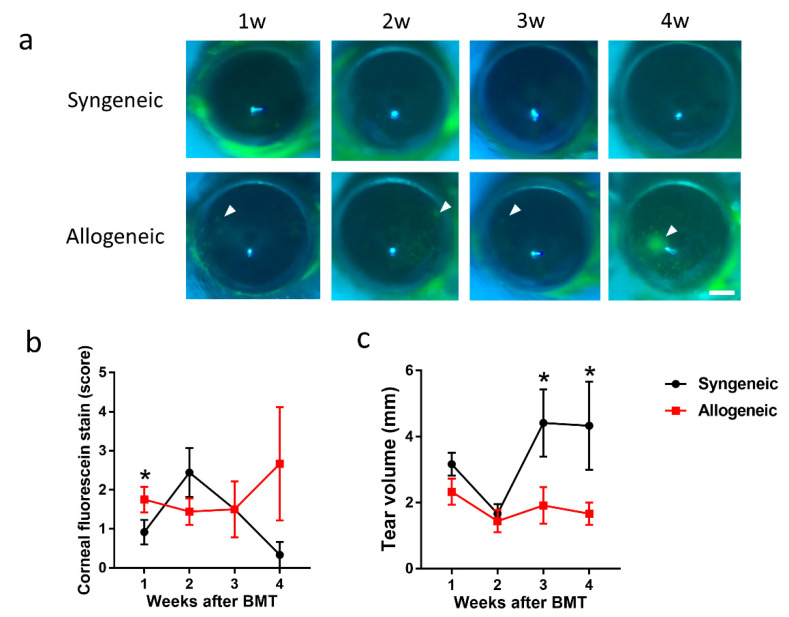
Serial changes in cornea of the syngeneic group and allogeneic group: (**a**) Representative images of corneal fluorescein staining and corneal erosion (arrowhead) Scale bar = 500 µm; (**b**) Corneal fluorescein staining score; (**c**) Tear volume. (*n* = 12 per group, 1week after BMT; *n* = 9 per group, 2 weeks after BMT; *n* = 6 per group, 3 weeks after BMT; *n* = 3 per group, 4 weeks after BMT). Data are presented as mean ± SEM. * *p* < 0.05, Mann-Whitney U Test.

Corneal epithelial damage was observed by corneal fluorescein staining (CFS) in both groups (Figure 5a). The CFS value was not significantly different in both groups 2 to 4 weeks after BMT (Figure 5b). However, the CFS value in the allogeneic group was significantly higher compared to the syngeneic group 1 week after BMT (Figure 5b). On the other hand, tear volume was significantly reduced through 1 to 2 weeks in both groups (Figure 5c). Two weeks after BMT, tear volume recovered in the syngeneic group but not in the allogeneic group (Figure 5c).

### 3.3. Histopathological Findings of Cornea and Lacrimal Gland in Syngeneic and Allogeneic Mice

In GVHD model mice, inflammatory findings were reported in the cornea [23,24] and fibro-inflammatory changes were found in the lacrimal gland [25]. We therefore focused on the cornea and lacrimal gland from syngeneic and allogeneic recipient mice to confirm that ocular GVHD had occurred in the mouse model.

**Figure 6:**
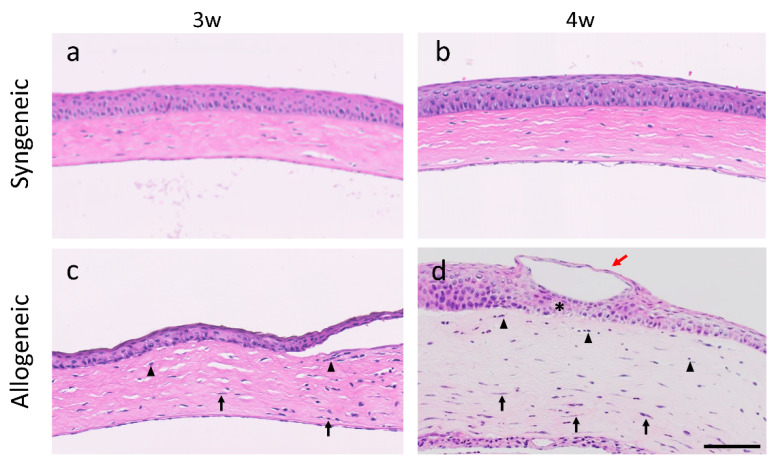
Hematoxylin & Eosin staining of cornea from the syngeneic and allogeneic mouse model: (**a**,**b**) Syngeneic group at 3 and 4 weeks after bone marrow transplantation; (**c**,**d**) Allogeneic group at 3 and at 4 weeks after bone marrow transplantation (black arrowhead, globular cells; black arrow, keratocytes; red arrow, epithelial erosion; *, abnormal regeneration of epithelia). Scale bar = 100 µm.

As for the cornea, HE staining revealed no obvious pathogenic or inflammatory changes in the tissue of the cornea in the syngeneic group (Figure 6a,b). However, the allogeneic group showed corneal edema, infiltration of globular cells, abnormal regeneration of epithelia, and an increase in number of keratocytes, especially under the area of the epithelial erosion 4 weeks after BMT (Figure 6c,d), similar to the confocal microscopic findings.

**Figure 7:**
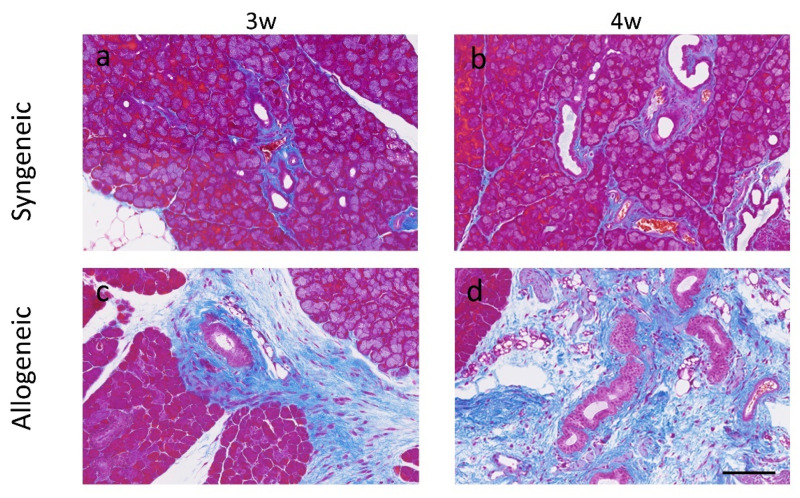
Mallory staining (blue) of lacrimal glands indicates fibrotic areas in the syngeneic and allogeneic mouse model: (**a**,**b**) Syngeneic group at 3 and 4 weeks after bone marrow transplantation; (**c**,**d**) Allogeneic group at 3 and at 4 weeks after bone marrow transplantation. Scale bar = 100 µm.

As for lacrimal glands, Mallory staining revealed no obvious pathological fibrotic and inflammatory changes in the syngeneic group (Figure 7a,b). However, in the allogeneic group, Mallory staining of lacrimal glands showed increased infiltration and pathological fibrosis surrounding the ducts 3 weeks after BMT, and the area was further expanded at 4 weeks after BMT (Figure 7c,d), suggesting that ocular GVHD developed in the cornea and lacrimal gland of this mouse model.

### 3.4. cGVHD Patients

**Figure 8:**
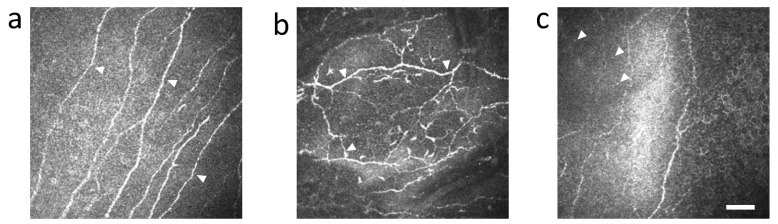
Representative IVCM images of cornea in HSCT recipients without GVHD (**a**) and with GVHD (**b**,**c**): (**a**) nerve fibers with no abnormality in HSCT recipients without GVHD (arrowhead); (**b**) nerve fibers with tortuosity and branching in HSCT recipients with GVHD (arrowhead); (**c**) small and globular cells in HSCT recipients with GVHD (arrowhead). Scale bar = 50 µm.

Corneas of HSCT recipients with and without GVHD were observed by IVCM. Corneal nerves of HSCT recipients without GVHD were straight and rarely branched (Figure 8a). On the other hand, corneal nerves of HSCT recipients with GVHD showed tortuosity and branching (Figure 8b). In addition, dendritic cells (Figure 8b) and small and globular cells (Figure 8c) were observed surrounding the abnormal nerves (Figure 8b,c).

## 4. Discussion

Ocular GVHD-related DED is the most common symptom of cGVHD, which often progresses to serious conditions that may lead to visual impairment [5,9,26]. It is therefore necessary to elucidate the pathological process involved in the onset and progression of GVHD. In this study, we showed how changes in cells, nerves, and neovascularization in the cornea of cGVHD mice observed by IVCM were very similar to IVCM observations in the cornea of ocular GVHD patients. IVCM is a powerful tool to non-invasively observe intracorneal cells and nerves in order to study the development of ocular GVHD and determine how these findings may be related to symptoms of ocular GVHD. Furthermore, the findings in IVCM images were corroborated by HE staining. The shape and location of globular cells and keratocytes in the cornea were similar between IVCM and HE staining. Therefore, not only can IVCM be used as a diagnostic tool, but it can also be used in animal experiments to study model animals non-invasively.

Acute corneal neovascularization may be a finding that predicts subsequent ocular GVHD in mice. In the early phase 1 week after BMT, neovascularization occurred, and epithelial damage and inflammatory cell infiltration were enhanced in all corneas of the allogeneic group (Figure 2 and Figure 4a, Supplementary Figure S1 and Video S1). This phenomenon has been reported for acute angiogenesis in intestinal GVHD [27], but it is the first to be reported in the cornea. Progenitor cells of donor-derived vascular endothelial cells infiltrate the intestinal tract and have been reported to cause angiogenesis early after BMT, and suppression of angiogenesis reduced the subsequent progression of GVHD [28,29]. We therefore speculate that suppression of angiogenesis may become a new therapeutic target for ocular GVHD.

The movement of cells observed by IVCM suggested an initial stage of inflammation in the cornea of cGVHD mice. Globular cell infiltration was significantly higher in the center of the cornea in the allogeneic group compared with the syngeneic group after BMT (Figure 3b). The increase of globular cells at 1 week after BMT in the allogeneic group may be associated with angiogenesis of the cornea. In other words, globular cells may have infiltrated the cornea through neovascularization. It is quite possible that the infiltration of globular cells in cornea triggers the onset of GVHD-related DED. Inflammatory cells release cytokines, and MMP9 released by corneal epithelial cells cause dysfunction of the tight junction in corneal epithelial cells. This in turn leads to loss of barrier function shown by fluorescein staining.

Dendritic cells were almost never observed at 1 week after BMT in the allogeneic group. Dendritic cells may have exited the cornea through lymphatic vessels to migrate to the cervical lymph node to prime inflammatory cells for subsequent immune response in the cornea. Interestingly, the number recovered by 2 weeks after BMT was similar to levels in the syngeneic group (Figure 3a). The results suggest that dendritic cells may have come to the cornea through lymph vessels. A similar mechanism of inflammatory cell migration to the cornea was reported by Dana, R et al. via applying external stimulus to the cornea in the form of dry air. Stimulated dendritic cells in the cornea then migrate from the corneal limbus to the cervical lymph nodes via lymph vessels, where naive T cells are then educated to differentiate into effector T cells such as Th1 and Th17 cells. Finally, effector T cells move to the cornea through the lymphatic vessels [30,31].

While we observed globular cells in the allogeneic group 4 weeks after BMT (Figure 4c) and in HSCT recipients with GVHD (Figure 8c), the identity of these cells needs to be determined. Since globular cells did not exist in normal corneas, we presume that they are inflammatory cells that contribute to the disease. Another common finding was tortuosity and branching of nerves observed in the allogeneic group 4 weeks after BMT and in HSCT recipients with GVHD. The tortuosity and branching of corneal nerves are thought to result from nerve regeneration caused by the repeated damage and release of nerve growth factor (NGF) and/or cytokines on ocular surface during inflammation in the cornea [32,33]. Thus, considering the similarity of IVCM images in the cornea of cGVHD model mouse and in cGVHD patients, it may be possible to predict the onset of cGVHD in HSCT recipients by careful observation of their corneas by IVCM following HSCT.

We investigated tear volume as a functional parameter of the lacrimal gland. Tear volume decreased at 2 weeks compared to 1 week after BMT in both the syngeneic group and the allogeneic group. This initial decrease in lacrimal gland function is probably due to radiation injury early in the course after BMT. However, at 3–4 weeks after BMT the tear volume was restored in the syngeneic group, whereas it was significantly decreased in the allogeneic group. The difference can be explained by pathological changes in the allogeneic group shown by inflammatory cell infiltration and pathological fibrosis of lacrimal glands 3–4 weeks after BMT (Figure 7a–d). There were no abnormal pathological findings in the syngeneic group. These finding suggest that the decreased tear volume due to impaired lacrimal gland function exacerbated corneal epithelial damage shown by increased corneal fluorescein scores (Figure 5a,b).

One of the limitations of our study is that we did not follow all the cGVHD mice for up to 4 weeks after BMT. This is due to the fact that GVHD mice often die when anesthetized, and corneal epithelial damage may occur due to the effects of anesthesia. We therefore analyzed different animals at 1–4weeks after BMT instead of analyzing the same animal sequentially. We hope to modify the anesthesia protocol so that the effect on the cornea is minimal allowing repeated serial analysis on the same mouse. Another limitation is that the sample size in this study was small due to the difficulty in preparing and breeding the GVHD mice simultaneously. We therefore needed to repeat the study several times. Furthermore, we could not identify cells in the cornea in detail since IVCM images were in gray scale and limited in resolution. It is generally accepted that the onset of cGVHD is caused by both the activation of antigen-presenting cells by irradiation prior to BMT, and tissue damage caused by anti-cancer agents and interacting with donor-derived T cells after BMT [34]. However, it is still unclear whether a similar phenomenon occurred in this study. In the future, we hope to clarify the identity of globular cells and dendritic cells by immunostaining, flow cytometry and pursue their role in the cervical lymph nodes. We also would like to clarify origin of the cells by observing the dynamics of donor-derived cells by using GFP mice as donors.

In conclusion, we reported the early pathophysiological changes in the cornea of an established cGVHD mouse model by IVCM. An NIH Consensus Conference suggested the importance of early diagnosis for GVHD [35], and many researchers have since focused on the ocular symptoms of GVHD [36]. Since IVCM is approved for clinical examination in patients, it should prove to be a powerful tool in the early diagnosis and treatment in order to prevent GVHD-related DED. Furthermore, IVCM images may become more important in analyzing the condition of the disease with the implementation of artificial intelligence [14]. GVHD-related DED is often recurrent and refractory, and irreversible damage may lead to blindness [11]. In the future, we hope to decrease the number of the patients by early detection of this intractable disease with IVCM image as a diagnostic tool.

## 5. Patents

Y.O. has a patent in Japan (patent no. 4966019; Name; Topical application and oral intake of tranilast for the treatment of chronic GVHD-related dye eye disease) and patent application number JP 2017018643 published as JPA2017-178922, application number JP2018-510646 published as WO2017/175808 and application number JP 2019-004730 published as JPA2020-111548.

## Data Availability

The data presented in this study are available upon reasonable request to the corresponding author.

## Supplementary Materials

The following are available online at https://www.mdpi.com/article/10.3390/diagnostics11081515/s1, Video S1: In vivo confocal microscopy video of neovascularization in cornea of the allogeneic group 1 week after bone marrow transplantation. Blood cells can be seen flowing through the vessels, Figure S1: Neovascularization in corneas of the allogeneic group 1 week after bone marrow trans-plantation shown by in vivo confocal microscopy (red circle). Scale bar = 500 µm.

## Abbreviations

GVHD	Graft-versus-host disease
HSCT	Hematopoietic stem cell transplantation
BMT	Bone marrow transplantation
NIH	National Institute of Health
IVCM	In vivo confocal microscopy
DED	Dry eye disease
RPMI	Roswell Park Memorial Institute
ACK	ammonium-chloride-potassium
HRT2-RCM	Heidelberg Retina Tomograph 2 Rostock Cornea Module
PMMA	poly-methyl-methacrylate
CFS	Corneal fluorescein staining
HE	Hematoxylin & Eosin
NGF	Nerve growth factor

## References

[1] Jagasia M.H., Greinix H.T., Arora M., Williams K.M., Wolff D., Cowen E.W., Palmer J., Weisdorf D., Treister N.S., Cheng G.S. (2015). National Institutes of Health Consensus Development Project on Criteria for Clinical Trials in Chronic Graft-versus-Host Disease: I. The 2014 Diagnosis and Staging Working Group report. Biol. Blood Marrow Transplant..

[2] Sato S., Shimizu E., He J., Ogawa M., Asai K., Yazu H., Rusch R., Yamane M., Yang F., Fukuda S. (2021). Positive Effects of Oral Antibiotic Administration in Murine Chronic Graft-Versus-Host Disease. Int. J. Mol. Sci..

[3] Hayashi S., Shimizu E., Uchino M., Yazu H., Aketa N., Tsubota K., Ogawa Y. (2020). The Overlap Syndrome: A Case Report of Chronic Graft-Versus-Host Disease after the Development of a Pseudomembrane. Cornea.

[4] Zeiser R., Blazar B.R. (2017). Pathophysiology of Chronic Graft-versus-Host Disease and Therapeutic Targets. N. Engl. J. Med..

[5] Filipovich A.H., Weisdorf D., Pavletic S., Socie G., Wingard J.R., Lee S.J., Martin P., Chien J., Przepiorka D., Couriel D. (2005). National Institutes of Health consensus development project on criteria for clinical trials in chronic graft-versus-host disease: I. Diagnosis and staging working group report. Biol. Blood Marrow Transplant..

[6] Ogawa Y., Okamoto S., Wakui M., Watanabe R., Yamada M., Yoshino M., Ono M., Yang H.Y., Mashima Y., Oguchi Y. (1999). Dry eye after haematopoietic stem cell transplantation. Br. J. Ophthalmol..

[7] Shimizu E., Ogawa Y., Saijo Y., Yamane M., Uchino M., Kamoi M., Fukui M., Yang F., He J., Mukai S. (2019). Commensal microflora in human conjunctiva; characteristics of microflora in the patients with chronic ocular graft-versus-host disease. Ocul. Surf..

[8] Shimizu E., Aketa N., Yazu H., Uchino M., Kamoi M., Sato Y., Tsubota K., Ogawa Y. (2020). Corneal higher-order aberrations in eyes with chronic ocular graft-versus-host disease. Ocul. Surf..

[9] Inamoto Y., Valdés-Sanz N., Ogawa Y., Alves M., Berchicci L., Galvin J., Greinix H., Hale G.A., Horn B., Kelly D. (2019). Ocular graft-versus-host disease after hematopoietic cell transplantation: Expert review from the Late Effects and Quality of Life Working Committee of the CIBMTR and Transplant Complications Working Party of the EBMT. Bone Marrow Transplant..

[10] Uchino M., Ogawa Y., Uchino Y., Mori T., Okamoto S., Tsubota K. (2012). Comparison of stem cell sources in the severity of dry eye after allogeneic haematopoietic stem cell transplantation. Br. J. Ophthalmol..

[11] Shikari H., Antin J.H., Dana R. (2013). Ocular graft-versus-host disease: A review. Surv. Ophthalmol..

[12] Kobayashi A., Yoshita T., Sugiyama K. (2005). In vivo findings of the bulbar/palpebral conjunctiva and presumed meibomian glands by laser scanning confocal microscopy. Cornea.

[13] Sterenczak K.A., Stache N., Bohn S., Allgeier S., Köhler B., Bartschat A., George C., Guthoff R.F., Stachs O., Stachs A. (2021). Burst of Corneal Dendritic Cells during Trastuzumab and Paclitaxel Treatment. Diagnostics.

[14] Williams B.M., Borroni D., Liu R., Zhao Y., Zhang J., Lim J., Ma B., Romano V., Qi H., Ferdousi M. (2020). An artificial intelligence-based deep learning algorithm for the diagnosis of diabetic neuropathy using corneal confocal microscopy: A development and validation study. Diabetologia.

[15] He J., Ogawa Y., Mukai S., Saijo-Ban Y., Kamoi M., Uchino M., Yamane M., Ozawa N., Fukui M., Mori T. (2017). In Vivo Confocal Microscopy Evaluation of Ocular Surface with Graft-Versus-Host Disease-Related Dry Eye Disease. Sci. Rep..

[16] Zhang Y., McCormick L.L., Desai S.R., Wu C., Gilliam A.C. (2002). Murine sclerodermatous graft-versus-host disease, a model for human scleroderma: Cutaneous cytokines, chemokines, and immune cell activation. J. Immunol..

[17] Shimizu E., Ogawa Y., Yazu H., Aketa N., Yang F., Yamane M., Sato Y., Kawakami Y., Tsubota K. (2019). “Smart Eye Camera”: An innovative technique to evaluate tear film breakup time in a murine dry eye disease model. PLoS ONE.

[18] Sung M.S., Li Z., Cui L., Choi J.S., Choi W., Park M.J., Park S.H., Yoon K.C. (2015). Effect of Topical 5-Aminoimidazole-4-carboxamide-1-β-d-Ribofuranoside in a Mouse Model of Experimental Dry Eye. Investig. Ophthalmol. Vis. Sci..

[19] Lin Z., Liu X., Zhou T., Wang Y., Bai L., He H., Liu Z. (2011). A mouse dry eye model induced by topical administration of benzalkonium chloride. Mol. Vis..

[20] Yaguchi S., Ogawa Y., Shimmura S., Hatou S., Nakamura S., Inaba T., Imada T., Ozawa Y., Kawakami Y., Ishida S. (2012). Presence and physiologic function of the renin-angiotensin system in mouse lacrimal gland. Investig. Ophthalmol. Vis. Sci..

[21] Ogawa Y., Morikawa S., Okano H., Mabuchi Y., Suzuki S., Yaguchi T., Sato Y., Mukai S., Yaguchi S., Inaba T. (2016). MHC-compatible bone marrow stromal/stem cells trigger fibrosis by activating host T cells in a scleroderma mouse model. eLife.

[22] Yaguchi S., Ogawa Y., Shimmura S., Kawakita T., Hatou S., Satofuka S., Nakamura S., Imada T., Miyashita H., Yoshida S. (2013). Angiotensin II type 1 receptor antagonist attenuates lacrimal gland, lung, and liver fibrosis in a murine model of chronic graft-versus-host disease. PLoS ONE.

[23] Perez R.L., Perez-Simon J.A., Caballero-Velazquez T., Flores T., Carrancio S., Herrero C., Blanco B., Gutierrez-Cosio S., Canete-Campos C., Cruz Gonzalez F. (2011). Limbus damage in ocular graft-versus-host disease. Biol. Blood Marrow Transplant..

[24] Perez V.L., Barsam A., Duffort S., Urbieta M., Barreras H., Lightbourn C., Komanduri K.V., Levy R.B. (2016). Novel Scoring Criteria for the Evaluation of Ocular Graft-versus-Host Disease in a Preclinical Allogeneic Hematopoietic Stem Cell Transplantation Animal Model. Biol. Blood Marrow Transplant..

[25] Yamane M., Sato S., Shimizu E., Shibata S., Hayano M., Yaguchi T., Kamijuku H., Ogawa M., Suzuki T., Mukai S. (2020). Senescence-associated secretory phenotype promotes chronic ocular graft-vs-host disease in mice and humans. FASEB J. Off. Publ. Fed. Am. Soc. Exp. Biol..

[26] Ogawa Y., Kim S.K., Dana R., Clayton J., Jain S., Rosenblatt M.I., Perez V.L., Shikari H., Riemens A., Tsubota K. (2013). International Chronic Ocular Graft-vs-Host-Disease (GVHD) Consensus Group: Proposed diagnostic criteria for chronic GVHD (Part I). Sci. Rep..

[27] Riesner K., Shi Y., Jacobi A., Kräter M., Kalupa M., McGearey A., Mertlitz S., Cordes S., Schrezenmeier J.F., Mengwasser J. (2017). Initiation of acute graft-versus-host disease by angiogenesis. Blood.

[28] Penack O., Henke E., Suh D., King C.G., Smith O.M., Na I.K., Holland A.M., Ghosh A., Lu S.X., Jenq R.R. (2010). Inhibition of neovascularization to simultaneously ameliorate graft-vs-host disease and decrease tumor growth. J. Natl. Cancer Inst..

[29] Leonhardt F., Grundmann S., Behe M., Bluhm F., Dumont R.A., Braun F., Fani M., Riesner K., Prinz G., Hechinger A.K. (2013). Inflammatory neovascularization during graft-versus-host disease is regulated by alphav integrin and miR-100. Blood.

[30] Chauhan S.K., Dana R. (2009). Role of Th17 cells in the immunopathogenesis of dry eye disease. Mucosal. Immunol..

[31] Tsubota K., Pflugfelder S.C., Liu Z., Baudouin C., Kim H.M., Messmer E.M., Kruse F., Liang L., Carreno-Galeano J.T., Rolando M. (2020). Defining Dry Eye from a Clinical Perspective. Int. J. Mol. Sci..

[32] Zhang M., Chen J., Luo L., Xiao Q., Sun M., Liu Z. (2005). Altered corneal nerves in aqueous tear deficiency viewed by in vivo confocal microscopy. Cornea.

[33] Villani E., Galimberti D., Viola F., Mapelli C., Ratiglia R. (2007). The cornea in Sjogren’s syndrome: An in vivo confocal study. Investig. Ophthalmol. Vis. Sci..

[34] Ferrara J.L., Cooke K.R., Teshima T. (2003). The pathophysiology of acute graft-versus-host disease. Int. J. Hematol..

[35] Wolff D., Radojcic V., Lafyatis R., Cinar R., Rosenstein R.K., Cowen E.W., Cheng G.S., Sheshadri A., Bergeron A., Williams K.M. (2021). National Institutes of Health Consensus Development Project on Criteria for Clinical Trials in Chronic Graft-versus-Host Disease: IV. The 2020 Highly morbid forms report. Transplant. Cell. Ther..

[36] Kitko C.L., Pidala J., Schoemans H.M., Lawitschka A., Flowers M.E., Cowen E.W., Tkaczyk E., Farhadfar N., Jain S., Steven P. (2021). National Institutes of Health Consensus Development Project on Criteria for Clinical Trials in Chronic Graft-versus-Host Disease: IIa. The 2020 Clinical Implementation and Early Diagnosis Working Group Report. Transplant. Cell. Ther..

