# Supplementary material for: Observation of Chronic Graft-Versus-Host Disease Mouse Model Cornea with In Vivo Confocal Microscopy
Source: Diagnostics (Basel). 2021 Aug 23;11(8):1515. doi: 10.3390/diagnostics11081515 (PMC8394898; doi:10.3390/diagnostics11081515)
Supplement: Supplementary file 1 [file diagnostics-11-01515-s001.zip › Supplementary Video and Figure Legends.pdf]

Supplementary Video and Figure legends

Observation of Chronic Graft-Versus-Host Disease

Mouse Model Cornea with In Vivo Confocal Microscopy

Shota Shimizu [1], Shinri Sato [1,*], Hiroko Taniguchi [1], Eisuke Shimizu [1], Jingliang He [1,2], Shunsuke Hayashi [1], Kazuno Negishi [1], Yoko Ogawa [1,*] and Shigeto Shimmura [1]

[1] Department of Ophthalmology, Keio University School of Medicine, Tokyo 160-8582, Japan; shimisho@keio.jp (S.Shimizu.); shinri.sato259@keio.jp (S.Sato.); tani@keio.jp (H.T.); ophthalmolog1st.acek39@keio.jp (E.S.); shun.hayashi8840@keio.jp (S.H.);kazunonegishi@keio.jp (K.N.); yoko@z7.keio.jp (Y.O.); shige.shimmura@keio.jp (S.Shimmura)

[2] Eye center, The Second Affiliated Hospital of Zhejiang University School of Medicine, Hangzhou, China; hejingliangai@126.com (J.H.)

* Co-correspondence: shinri.sato259@keio.jp; Tel.: +81-3-3353-1211;(S. Sato);Tel.: +81-3-3353-1211; yoko@z7.keio.jp; Tel.: +81-3-3353-1211;(Y.O)

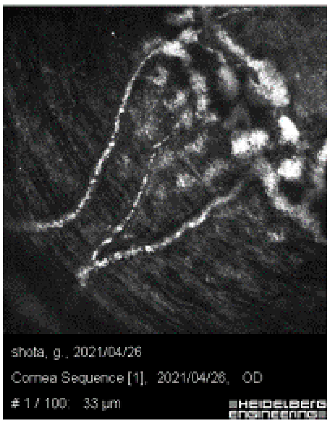

Supplementary Video S1. In vivo confocal microscopy video of neovascularization in cornea of the allogeneic group 1 week after bone marrow transplantation. Blood cells can be seen flowing through the vessels.

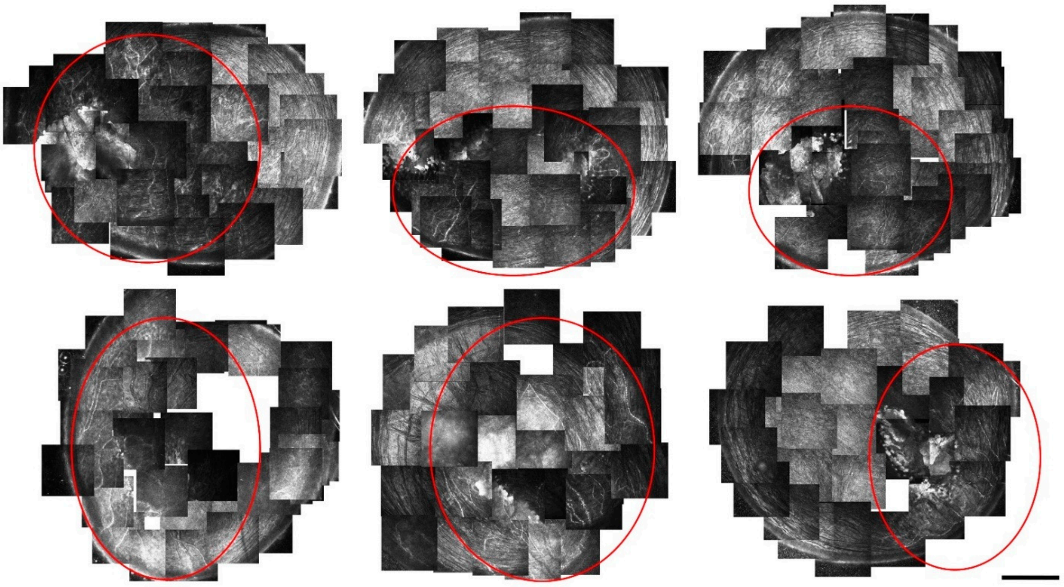

Supplementary Figure S1. Neovascularization in corneas of the allogeneic group 1 week after bone marrow transplantation shown by in vivo confocal microscopy (red circle). Scale bar = 500 μm.
