# Supplementary figures and images for: Observation of Chronic Graft-Versus-Host Disease Mouse Model Cornea with In Vivo Confocal Microscopy
Source: Diagnostics (Basel). 2021 Aug 23;11(8):1515. doi: 10.3390/diagnostics11081515 (PMC8394898; doi:10.3390/diagnostics11081515)

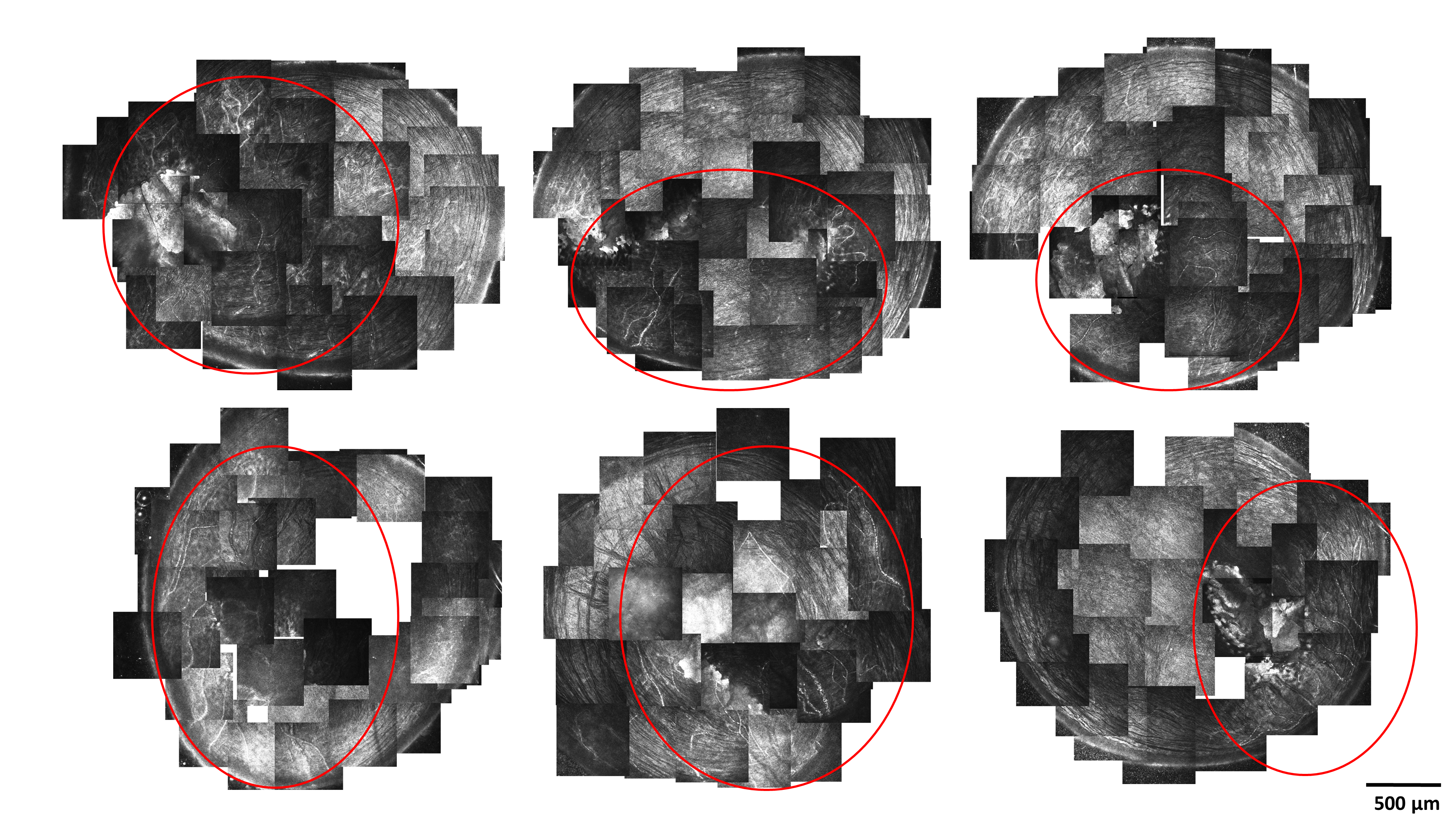

Supplement: Supplementary file 1 [file diagnostics-11-01515-s001.zip › Supplementary Figure S1.tif]
